# Multilevel posterior spinal fusion following percutaneous third generation kyphoplasty complicated by vertebral compression fracture progression

**DOI:** 10.1093/jscr/rjae193

**Published:** 2024-03-27

**Authors:** Bongseok Jung, Anas Abbas, Justin Han, Alex Ngan, Austen Katz, David Essig

**Affiliations:** Department of Orthopaedic Spine Surgery, Northwell Health Long Island Jewish Medical Center, New Hyde Park, NY 11040, United States; Department of Orthopaedic Spine Surgery, Northwell Health Long Island Jewish Medical Center, New Hyde Park, NY 11040, United States; Department of Orthopaedic Spine Surgery, Northwell Health Long Island Jewish Medical Center, New Hyde Park, NY 11040, United States; Department of Orthopaedic Spine Surgery, Northwell Health Long Island Jewish Medical Center, New Hyde Park, NY 11040, United States; Department of Orthopaedic Spine Surgery, Northwell Health Long Island Jewish Medical Center, New Hyde Park, NY 11040, United States; Department of Orthopaedic Spine Surgery, Northwell Health Long Island Jewish Medical Center, New Hyde Park, NY 11040, United States

**Keywords:** kyphoplasty, fusion, vertebra, compression fracture, lumbar, laminectomy

## Abstract

Newer third generation percutaneous kyphoplasty (PKs) may minimize risks associated with older generation kyphoplasties such as new adjacent fractures, fracture progression, cement leakage, neurologic sequelae, and kyphosis. Additionally, posterior pedicle spinal fusion (PPSF) may minimize risk of long-term complications following PKs while maximizing the benefits of stable spinal alignment. The patient developed adjacent fracture progression, posterior retropulsion, and kyphosis following third generation kyphoplasty. Vertebral compression fracture progression was corrected and prolonged symptomatic relief was successfully achieved with T11-L4 PPSF and L1-L2 laminectomy. Postoperative follow-ups at 2, 4, 7 weeks, 1 and 2 years showed continued symptomatic improvement in back pain with resolution of thigh and groin pain. This case supports the use of PPSF in third generation PK-related complications to provide long-term symptom relief and improve quality of life in patients with severe osteoporotic compression fractures.

## Teaser

Multilevel posterior pedicle spinal fusion to correct complications of refracture, posterior retropulsion, and kyphosis following third generation kyphoplasty.

## Introduction

Vertebral compression fractures are a growing public health concern. They have an estimated prevalence of 30–50% in people over 50 years of age, with a substantial economic burden estimated at 13–18 billion US dollars annually [1, 2]. Vertebral augmentation through percutaneous kyphoplasty (PK) has been shown to be an effective, standard interventional procedure for many osteoporotic vertebral compression fracture (OVCF) cases [3]. However, complications may be associated with these procedures including new adjacent vertebral fracture, cement leakage, back pain, kyphosis, infection, and fracture progression [4].

Posterior pedicle spinal fusion (PPSF) may be an effective treatment option for patients suffering complications from vertebral augmentation. However, there are few reported cases of surgery for the spinal complications of third generation PK. Furthermore, there is a scarcity of studies regarding PPSF in third generation PK patients who developed vertebrae fracture progression. Here, we present a case of multilevel PPSF that was performed for a patient who developed L2 vertebral fracture progression 2 weeks post-third generation PK with neurologic symptoms and deformity.

## Case presentation

**Figure 1:**
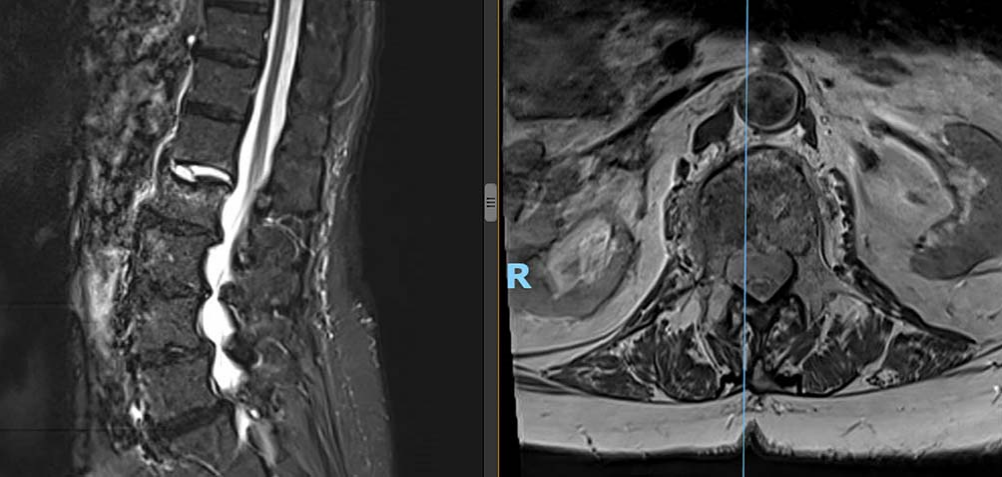
Preoperative lateral (left) and axial (right) MRI of the patient’s spine showing L2 compression fracture.

The patient is an 82-year-old female with a history of hypertension and osteoporosis with BMI of 34.39. The patient presented with worsening, 10/10 lower back pain for the past 3 weeks, which interfered with her ability to perform activities of daily living (ADLs). Her pain started in the midline lumbar spine, radiating laterally across her back into her buttocks and bilateral groin. MRI with no contrast of the lumbar spine revealed L2 compression fracture with concurrent injury to the L1-L2 disk (Fig. 1). The patient was referred to interventional radiology (IR) for L2 kyphoplasty.

**Figure 2:**
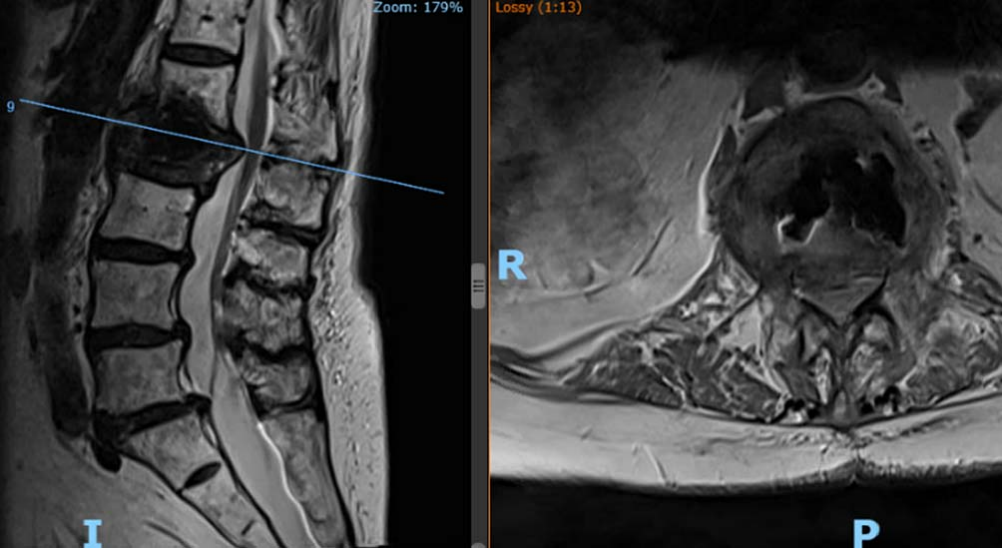
Patient’s lateral (left) and axial (right) MRI following the kyphoplasty procedure, demonstrating L1 fracture with retropulsion and compression of the conus.

Fluoroscopic guided vertebral augmentation with cavity creation and implant implantation was successfully performed on the L2 vertebral body by IR (Fig. 2). Pedicle access was achieved through a bilateral transpedicular approach, and the cavity was accessed through an 11-gauge trocar. The implant device used a 5.8 mm Spine Jack® (Stryker Corp, Kalamazoo, MI) bilaterally and the cement injected into the cavity was synthetic resin polymethyl methacrylate (PNMA). There were no clinically significant cement leak occurrences during or after the kyphoplasty procedure.

**Figure 3:**
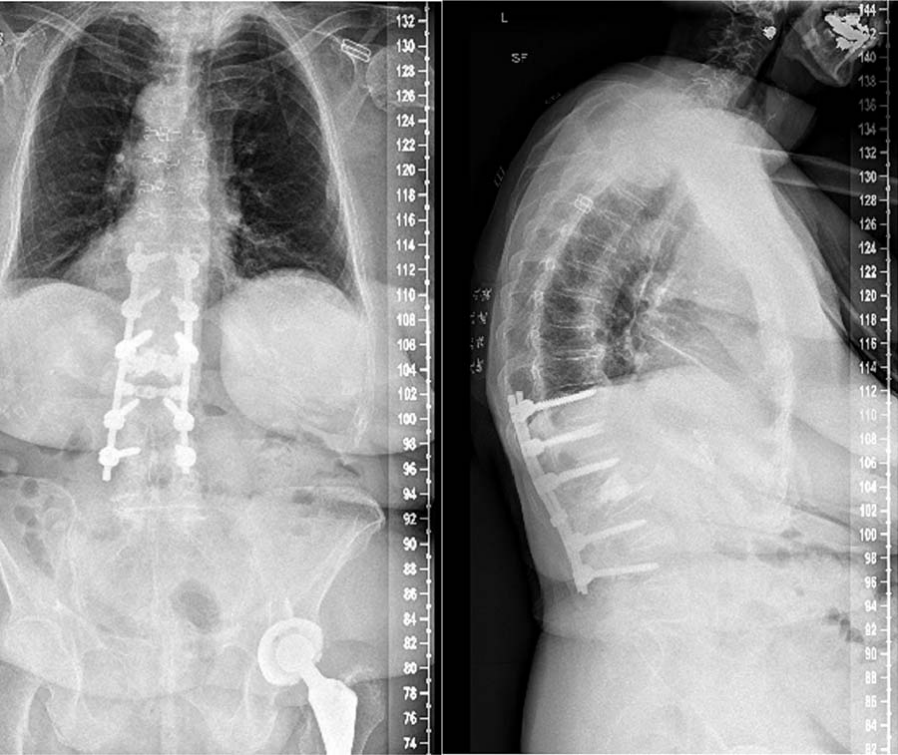
Anterior–posterior and lateral radiographs of the patient’s spine postoperatively, following L1-L2 laminectomy and T11-L4 posterior fusion.

Two-week postoperative check revealed the patient was still feeling disabled, with a new onset of burning pain in the right groin and thigh, severe back soreness, numbness, and leg weakness, which were refractory to pain medications. Lumbosacral spine MRI redemonstrated the cemented L2 compression fracture with posterior bony retropulsion, along with progressive collapse of the anterior inferior corner of L1 impacted into the anterior aspect of the cemented L2 compression fracture, associated kyphosis at L2, and multilevel degenerative disk changes (Fig. 3). After an extensive review of possible management options for the patient’s symptoms, the patient elected to undergo an open-approach multilevel PPSF.

Segmental spinal instrumentation was placed at L4, L3, L1, T12, and T11 with appropriate positioning of the screws confirmed with biplanar fluoroscopy. Laminectomy was also performed at L2 and L1 with adequate decompression of the neural elements. The fracture was thereafter reduced using gentle cantilever maneuvers. One crosslink was placed, and a final fluoroscopic image confirmed the appropriate positioning of implants and reduction of the fracture and deformity. The wound was copiously irrigated, and the posterolateral spine was decorticated and packed with local autograft as well as demineralized bone matrix. Plastic surgery ensured proper closure using muscle flap reconstruction of the patient’s posterior spine wound. Follow-ups at 2, 4, and 7 weeks later all revealed that the patient no longer had stabbing or throbbing pain, gradual improvement of the back pain down to a 3/10 in severity, well-tolerated ambulation with a walker and assist, normal CNII-VII, 5/5 strength bilaterally upper and lower extremities, and mild back soreness. Postoperative follow-up at 2 years still revealed no complications associated with the procedure with the patient having minimal back pain and soreness, supported by lateral and axial radiographs (Fig. 3).

## Discussion

Third generation kyphoplasty indefinitely restores vertebral height using an expandable scaffold through a mechanical rather than hydraulic opening control prior to injection of the cement. Due to the implantation of a permanent intervertebral scaffold for height maintenance, studies have shown that third generation PK are associated with lower rates of new fractures and a larger postoperative increase in vertebral heights when compared with traditional kyphoplasty [5, 6]. Spine Jack® (Stryker Corp, Kalamazoo, MI) specifically may be associated with reduced cement leakage and adjacent vertebral body fractures, along with reduction of the superior endplates allowing for better recovery of the injured disk [7].

There is a myriad of known potential risk factors associated with PK complications including bone mineral density, cement distribution, intradiscal cement leakage, and height restoration [8]. This case report also highlights a less reported but another potential risk factor of third-generation PK, adjacent discal injury.

Although the statistics vary across studies, an estimated 10% of the same operated vertebra may be refractured following PK, and at least 18–25% of patients may develop new symptomatic vertebral fractures after PK or PV [9, 10]. Limitations of these procedures are highlighted especially in the case of very severe OVCFs, defined as the reduction of more than two-thirds vertebral height, where cement augmentation alone may not be sufficient to correct severe kyphosis and maintain long-term sagittal balance [11]. Furthermore, in the case of vertebral compression fractures with posterior wall ruptures, kyphoplasty alone is contraindicated in clinical practice [12].

When performed independently, PK may reduce OVCF-related pain and restore vertebral height. On the other hand, PPSF provides long-lasting vertebral stabilization and improved capacity for kyphosis correction. Performing PPSF and PK in tandem has been suggested to provide superior kyphosis correction and long-term sagittal balance while reducing the risk of complications such as adjacent vertebral fractures [13]. Our report, in support of prior studies, suggests that PPSF may also effectively reduce OVCF-related pain and disability following post-kyphoplasty complications such as new adjacent vertebral fractures and kyphosis. Future studies should be directed toward identifying which fracture patterns and patient characteristics are best with stand-alone vertebral augmentation versus supplemental posterior pedicle screw fixation.
